# Dentoalveolar mandibular changes with self-ligating *versus* conventional bracket systems: A CBCT and dental cast study

**DOI:** 10.1590/2176-9451.20.3.050-057.oar

**Published:** 2015

**Authors:** Marcio Rodrigues de Almeida, Cristina Futagami, Ana Cláudia de Castro Ferreira Conti, Paula Vanessa Pedron Oltramari-Navarro, Ricardo de Lima Navarro

**Affiliations:** 1 Full professor of Orthodontics, Universidade Norte do Paraná (UNOPAR), Londrina, Paraná, Brazil; 2 MSc in Orthodontics, Universidade Norte do Paraná (UNOPAR), Londrina, Paraná, Brazil; 3 Professor of Orthodontics, Universidade do Sagrado Coração (USC), Bauru, São Paulo, Brazil; 4 PhD in Orthodontics, Universidade de São Paulo (USP), São Paulo, São Paulo, Brazil

**Keywords:** Orthodontic appliances, Corrective orthodontics, Orthodontic brackets

## Abstract

**OBJECTIVE:**

The aim of the present study was to compare dentoalveolar changes in mandibular arch, regarding transversal measures and buccal bone thickness, in patients undergoing the initial phase of orthodontic treatment with self-ligating or conventional bracket systems.

**METHODS:**

A sample of 25 patients requiring orthodontic treatment was assessed based on the bracket type. Group 1 comprised 13 patients bonded with 0.022-in self-ligating brackets (SLB). Group 2 included 12 patients bonded with 0.022-in conventional brackets (CLB). Cone-beam computed tomography (CBCT) scans and a 3D program (Dolphin) assessed changes in transversal width of buccal bone (TWBB) and buccal bone thickness (BBT) before (T_1_) and 7 months after treatment onset (T_2_). Measurements on dental casts were performed using a digital caliper. Differences between and within groups were analyzed by Student's t-test; Pearson correlation coefficient was also calculated.

**RESULTS:**

Significant mandibular expansion was observed for both groups; however, no significant differences were found between groups. There was significant decrease in mandibular buccal bone thickness and transversal width of buccal bone in both groups. There was no significant correlation between buccal bone thickness and dental arch expansion.

**CONCLUSIONS:**

There were no significant differences between self-ligating brackets and conventional brackets systems regarding mandibular arch expansion and changes in buccal bone thickness or transversal width of buccal bone.

## Abstract (pt)

**OBJETIVO:**

o objetivo do presente estudo foi comparar as alterações dentoalveolares transversais e a espessura óssea da arcada inferior em pacientes submetidos ao tratamento ortodôntico utilizando sistemas de braquetes autoligáveis ou convencionais.

**MÉTODOS:**

uma amostra de 25 pacientes requerendo tratamento ortodôntico foi recrutada com base no tipo de braquete. No Grupo 1, 13 pacientes foram tratados com braquetes autoligáveis (SLB, *slot* 0,022"); o Grupo 2 incluiu 12 pacientes, nos quais foram colados braquetes convencionais (CLB, *slot*0,022"). Utilizou-se tomografia computadorizada de feixe cônico e um programa 3D (Dolphin) para avaliar as alterações pré-tratamento (T_1_) e 7 meses após o início desse (T_2_). As medições em modelos de gesso foram realizadas com o auxílio de um paquímetro digital. As diferenças intergrupos, bem como intragrupo, foram analisadas por meio de teste *t* de Student. Além disso, o coeficiente de correlação de Pearson foi utilizado.

**RESULTADOS:**

alterações dentoalveolares significativas foram observadas em ambos os grupos. Entretanto, não houve diferenças significativas entre os grupos. Houve uma diminuição da espessura óssea na região posterior e das medidas transversais em ambos os grupos. Não houve uma correlação significativa entre a espessura óssea posterior e a expansão da arcada dentária, em nenhum dos dois sistemas de braquetes utilizados.

**CONCLUSÕES:**

comparando-se o uso dos aparelhos autoligáveis e convencionais, concluiu-se que não houve diferenças dentoalveolares significativas quanto à expansão da arcada inferior e quanto à espessura óssea posterior.

## INTRODUCTION

The ongoing search for innovation in Orthodontics has boosted the emergence or re-emergence of appliances so as to offer patients more comfort, shorter treatment time, improved post-treatment stability, and fewer side effects. Self-ligating brackets (SLB) came back into scene in the seventies, arising strong expectancy, and became popular in the nineties. Much empirical and anecdotal evidence as well as advantages were attributed to these appliances: increased patient comfort, better oral hygiene, increased patient cooperation, less chair time, shorter treatment time, greater patient acceptance, expansion, and less dental extractions.^1^^-^ 6

Correcting dental crowding without extractions or interproximal reductions requires an increase in arch perimeter in order to allow excellent teeth alignment. In the absence of distal movements, the dimensional changes of the arch involve transversal and buccal dental expansion.^7^ It is a well-known fact that both self-ligating and conventional ligating brackets (CLB) when used for non-extraction treatment of dental crowding produce dentoalveolar expansion. The amount of transversal increase depends on the mechanics applied in each case.^7^^-^ 11

Before the introduction of computerized tomography, it was not possible to visualize the buccal bone due to superposition that occurred in 2D radiographs.^12,13^ To achieve successful orthodontic treatment, the limits of orthodontic movement must be respected, in order to prevent iatrogenic effects to the sustaining and protection periodontium, such as gingival recessions, dehiscence and bone fenestrations. Studies prior to cone-beam computed tomography (CBCT) scans assessed only radiographs and dental casts, both of which used to be regarded as gold standards. Improvements in CBCT scans revealed it to be a reliable method, which offers an excellent visualization of the actual structures.^14,15^ Timock *et al* 16 investigated the accuracy and reproducibility of measurements of alveolar bone height and thickness by means of CBCT imaging. They found good precision and accuracy for both measurements.^16^

The transversal response of the mandibular dental arch treated with CLB has been widely studied in the literature, especially the dentoalveolar response on dental casts.^7,10,17,18^ However, little is known regarding CBCT scans used to assess the mandibular alveolar bone of the posterior region, where buccal bone can be detected and quantified.^19^ This study aims at testing the null hypothesis that there is no difference, regarding changes in transversal width and buccal bone thickness in the mandibular arch, between patients undergoing the initial phase of orthodontic treatment (7 months) with SLB and CLB systems. 

## MATERIAL AND METHODS

This research protocol was approved by Universidade Norte do Paraná (UNOPAR, Londrina/PR, Brazil) Institutional Review Board. Patients and guardians were fully informed about the study and its implications, and signed a consent form.

For this prospective study, power analysis showed that a sample size of 12 patients in each group would give 80% probability to detect a real difference of 1.4 mm in intermolar distance and 0.2 mm in bone thickness, with a 95% (p < 0.05) significance level.^20^ The sample for the present prospective randomized study was treated at Universidade Norte do Paraná (UNOPAR, Londrina, PR, Brazil) from 2009 to 2012. All patients had complete orthodontic records taken at the beginning (T_1_) of treatment and 7 months after treatment onset (T_2_), including study models and CBCT scans. In selecting the sample, the following inclusion criteria were applied: patients with Angle Class I malocclusion, moderate-to-severe lower dental crowding (3.0 to 7.0 mm), absence of diastema, absence of posterior crossbite, complete permanent teeth (except for third molars). Patients were randomly divided into two groups: SLB and CLB. Out of the selected individuals, none were excluded after treatment onset. Premolars extraction and tooth wear were not included in the proposed treatment.

Group 1 (G1) comprised 13 patients treated with 0.022 x 0.027-in slot SLB (EasyClip Aditek, Cravinhos/SP, Brazil), with initial mean age of 18.58 years (SD = 5.43). Group 2 (G2) comprised 12 patients treated with 0.022 x 0.028-in slot CLB (3M Unitek, Monrovia, Calif., USA), with initial mean age of 21.61 years (SD = 6.69). The archwires for Group 2 were tied to the brackets by means of a metallic ligature. Patients were orthodontically treated during initial leveling and alignment for six months, following the same sequence of round archwires: 0.013, 0.014 and 0.016-in nickel-titanium archwires, according to the manufacturers's (Aditek) prescription (Damon system). Each archwire remained in place for two months.

Cone-beam computed tomography scans were obtained from all patients at two time intervals prior to orthodontic treatment onset and 7 months after it. All CBCT scans were carried out by a single experienced radiologist using the same scanner (i-Cat Imaging Sciences International, Hatfield, Pennsylvania, USA) set up as follows: 22 x 16 cm fov, 40 sec, 120 kVp, 36 mA. This scanner has high-resolution sensors and affords 0.4-mm voxel images.^21^

CBCT scans were analyzed by one single operator who assessed mandibular bone changes by means of Dolphin 3D software (Version 11.5^(r)^, Dolphin Imaging & Management Solutions, Chatsworth, Calif., USA) with a level of sensitivity set at 25%.

**Figure 1.**
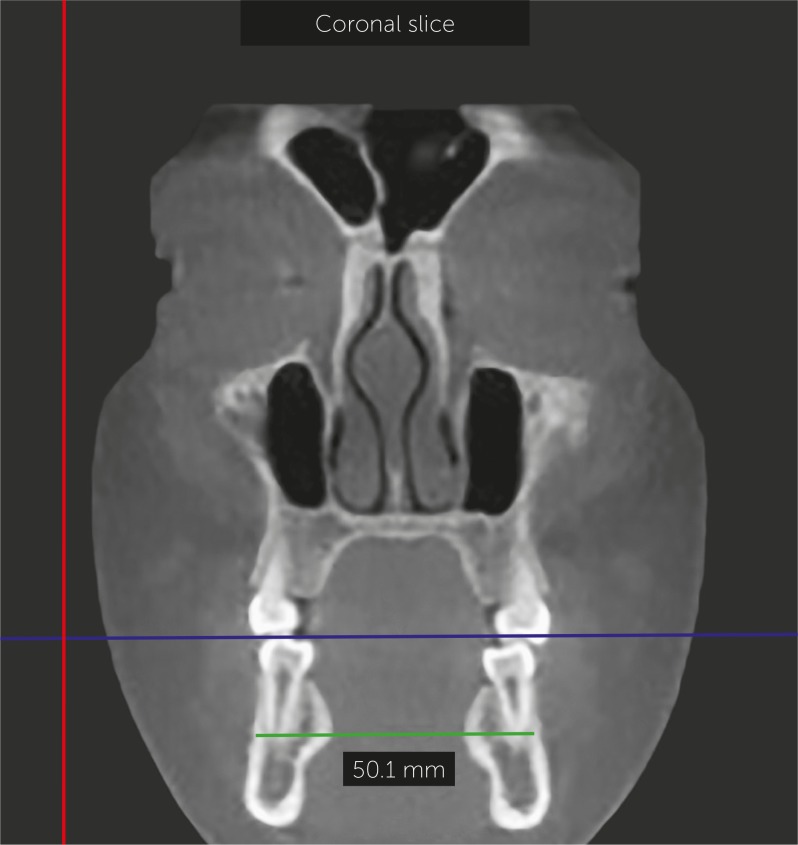
Coronal slice and transversal width of buccal bone (TWBB).

**Figure 2.**
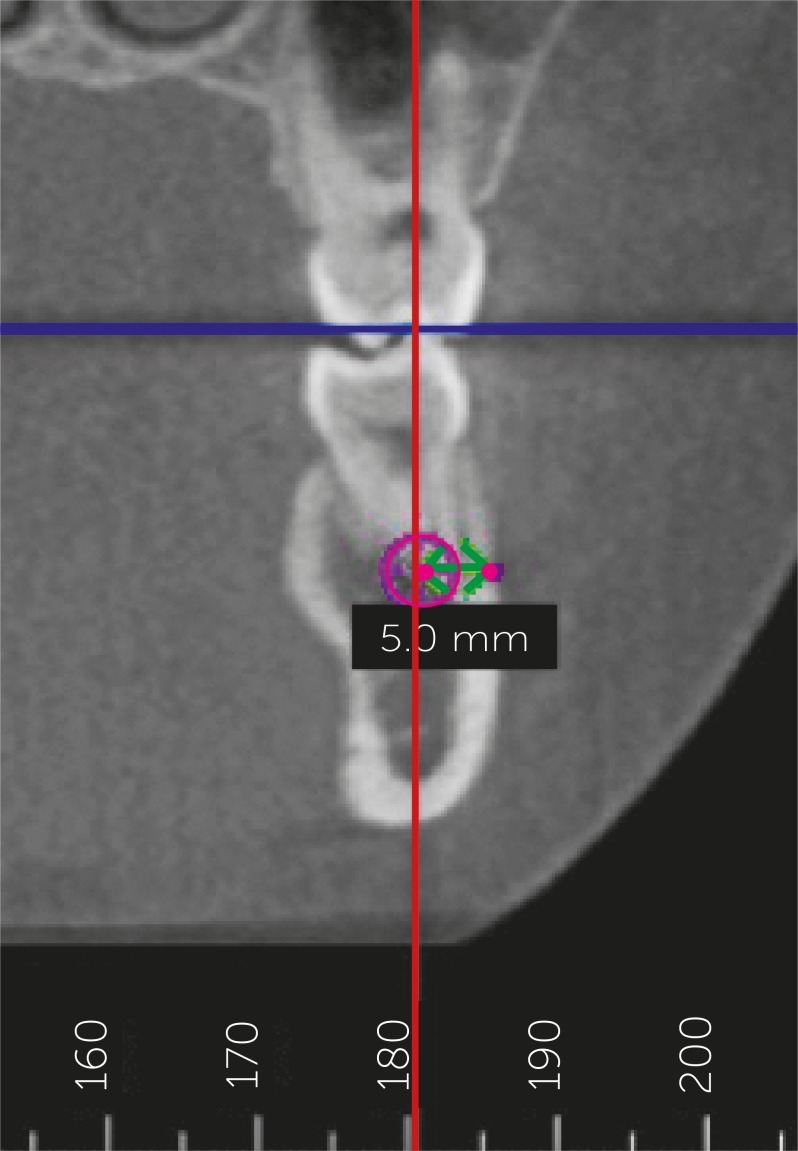
Buccal bone thickness (BBT) measurements.

**Figure 3.**
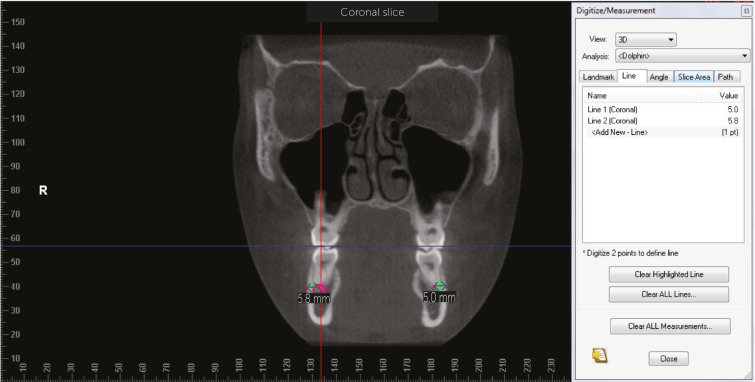
Example of measurements for buccal bone thickness (BBT) of mandibular first molars.

Coronal slices were selected for the bone measurements (Fig 1) and 1-mm thick cross-sections were made through the first molar (M1), second premolar (P2) and first premolar (P1), in the right and left mandibular arches. As for coronal slices, the mid portion of teeth (molars and premolars) was chosen. The point selected for buccal bone measurement was the most external prominence of the buccal bone (EBB) in the root most apical portion (apex). At this same height, a point was projected from the parallel projection of the cusp point. The distance between the two points was determined as BBT, buccal bone thickness (Figs 2 and 3). Thus, changes in BBT were calculated by subtracting T_1_ from T_2_ values. For transversal width of the buccal bone, the EBB point was used on the right and left sides. The distance between right and left EBB was the transversal width of buccal bone (TWBB) (Fig 1). Similarly, TWBB changes were calculated by subtracting T_1_ from T_2 _values. In order to confirm whether transversal width and bone thickness measurements were taken on the same coronal slices, the mid region of each posterior teeth was used as reference to ensure consistency of slices.

**Figure 4.**
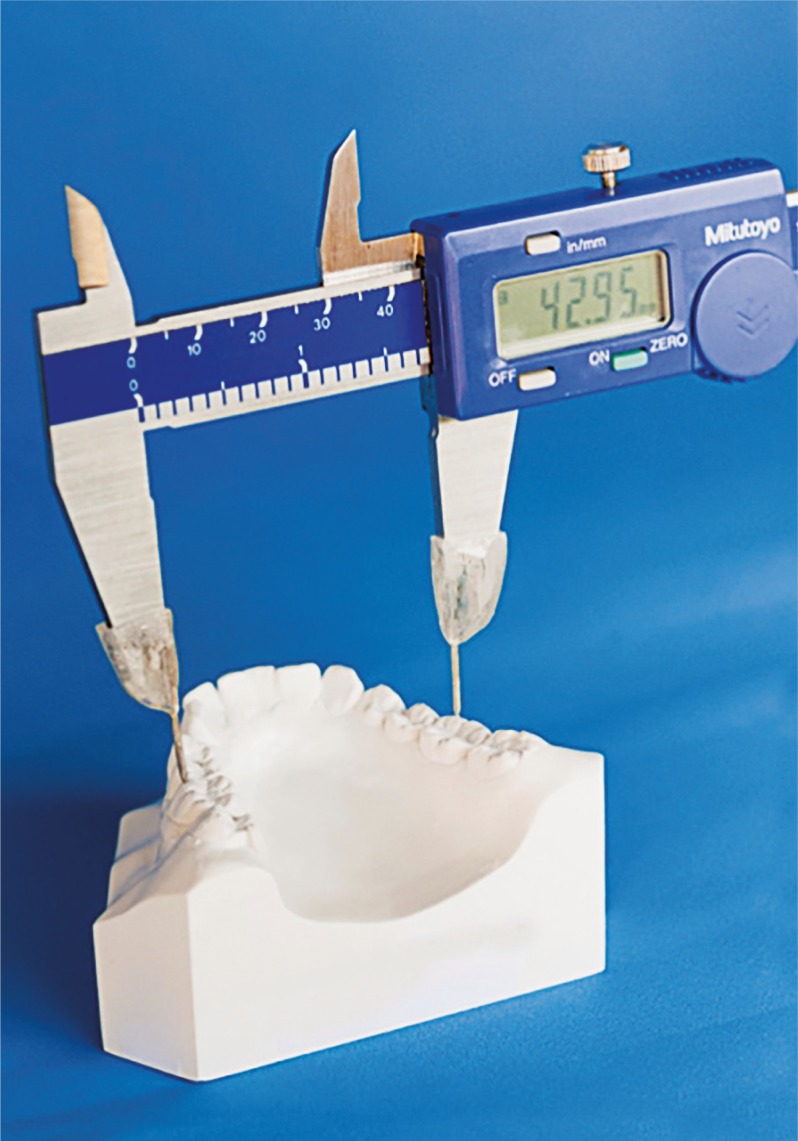
Intermolar width measured on a dental cast by means of a digital caliper.

Intermolar distances, intersecond premolar distances, and interfirst premolar distances were measured in dental casts (Fig 4) by means of a previously calibrated digital caliper (Mitutoyo Caliper, Japan). In order to measure the transversal distances, buccal cusp tips were selected for first and second mandibular premolars, while mesiobuccal cusp tips were selected for first molars.

## Statistical analysis

To assess intra and interexaminer reliability, ten CBCT scans were randomly selected and remeasured four weeks apart by two operators. Intraexaminer error was assessed by means of paired* t-*test and Dahlberg's formula. Interexaminer reliability was assessed by intraclass correlation coefficient (ICC). Data were tested for normal distribution by means of Kolmogorov-Smirnov test. As data were normally distributed, parametric tests were applied. Results were described by parameters of mean and standard deviation of T_1_ and T_2_ measurements for both groups. Independent *t-*tests were used to compare the initial demographic data of both groups. Paired and unpaired *t*-tests were used to compare intra and intergroup changes. Finally, Pearson correlation coefficient was calculated to further explore the association between dental expansion and expansion of TWBB. In all statistical tests, the significance level was set at 5%.^22^ All statistical analyses were performed with SPSS software for Windows version 17.0 (SPSS Inc, Chicago Ill.).

## RESULTS

Systematic (paired *t-*test*) *and casual error (Dahlberg's formula) showed no intraexaminer difference. Intraclass correlation coefficients for bone thickness and transversal width of buccal bone measurements were 0.89 and 0.98, respectively, thereby showing acceptable reliability. Random error ranged from 0.30 to 0.56 mm and from 0.53 to 1.08 mm for dental casts and CBCT measurements, respectively.

Patients' demographic distribution is presented in Table 1. Both samples were comparable at treatment onset regarding the following aspects: initial age, treatment time, intermolar distances, intersecond premolar distances, interfirst premolar distances, TWBB and BBT measurements. Means and standard deviation values for BBT and TWBB measurements at pretreatment (T_1_), 7 months after treatment onset (T_2_) and the changes observed (T_2_-T_1_) are shown in Tables 2 to 4.

**Table 1.** Patients' demographic distribution

	SLB (G1) (n = 13)	CLB (G2) (n = 12)	P
Initial mean age (years)	18.58 ± 5.43	21.61 ± 6.69	0.221
Treatment time (days)	210.15 ± 41.44	218.17 ± 46.60	0.654
CBCT scans			
P1L BBT (mm)	2.34 ± 2.59	2.31 ± 1.32	0.972
P1R BBT (mm)	2.65 ± 2.23	2.44 ± 1.21	0.788
P2L BBT (mm)	4.64 ± 2.38	4.02 ± 3.56	0.613
P2R BBT (mm)	4.82 ± 2.71	4.92 ± 2.11	0.927
M1L BBT (mm)	6.24 ± 2.29	6.49 ± 2.10	0.783
M1R BBT (mm)	6.70 ± 2.78	6.87 ± 1.48	0.856
P1 TWBB (mm)	40.37 ± 2.43	38.94 ± 2.58	0.175
P2 TWBB (mm)	49.35 ± 4.44	49.17 ± 4.07	0.928
M1 TWBB (mm)	59.04 ± 4.86	59.16 ± 4.45	0.956
Dental cast measurements (mm)			
4-4 width (mm)	33.95 ± 1.87	33.37 ± 2.36	0.749
5-5 width (mm)	38.42 ± 2.18	38.57 ± 2.69	0.888
6-6 width (mm)	44.85 ± 1.68	44.37 ± 2.76	0.612

M1 = first molar, P2 = second premolar and P1 = first premolar.

**Table 2.** Mean and standard deviation at the beginning of treatment (T1) and 7 months after treatment onset (T2), regarding changes in buccal bone thickness and transversal width of buccal bone (CBCT measurements) for the CLB group

Measurements	T_1_	T_2_	Diff.	P value
P1L BBT (mm)	2.31 ± 1.32	0.80 ± 1.86	-1.51	0.016*
P1R BBT (mm)	2.44 ± 1.21	1.54 ± 1.46	-0.90	0.039*
P1 TWBB (mm)	38.94 ± 2.58	38.73 ± 2.88	-0.21	0.613
P2L BBT (mm)	4.02 ± 3.56	3.14 ± 2.31	-0.88	0.165
P2R BBT (mm)	4.92 ± 2.11	3.83 ± 2.01	-1.09	0.007*
P2 TWBB (mm)	49.17 ± 4.07	48.52 ± 3.72	-0.66	0.222
M1L BBT (mm)	6.49 ± 2.10	6.18 ± 1.55	-0.31	0.292
M1R BBT (mm)	6.87 ± 1.48	6.08 ± 1.76	-0.79	0.008*
M1 TWBB (mm)	59.16 ± 4.45	58.90 ± 4.34	-0.26	0.611

* p < 0.05. M1 = first molar, P2 = second premolar and P1 = first premolar.

**Table 3.** Mean and standard deviation at the beginning of treatment (T1) and 7 months after treatment onset (T2), regarding changes in buccal bone thickness and transversal width of buccal bone (CBCT measurements) SLB group.

Measurements	T_1_	T_2_	Diff.	P value
P1L BBT (mm)	2.34 ± 2.59	1.69 ± 1.64	-0.66	0.177
P1R BBT (mm)	2.65 ± 2.23	1.77 ± 2.01	-0.88	0.019*
P1 TWBB (mm)	40.37 ± 2.43	39.82 ± 2.67	-0.56	0.076
P2L BBT (mm)	4.64 ± 2.38	4.00 ± 2.42	-0.64	0.002*
P2R BBT (mm)	4.82 ± 2.71	3.73 ± 2.40	-1.09	<0.001**
P2 TWBB (mm)	49.35 ± 4.44	49.34 ± 4.13	-0.01	0.980
M1R BBT (mm)	6.24 ± 2.29	5.93 ± 2.43	-0.32	0.158
M1R BBT (mm)	6.70 ± 2.78	6.16 ± 2.63	-0.54	0.025*
M1 TWBB (mm)	59.04 ± 4.86	58.94 ± 4.79	-0.10	0.750

* p < 0.05.

** p < 0.01. M1 = first molar, P2 = second premolar and P1 = first premolar.

**Table 4.** Means and standard deviation at the beginning of treatment (T1) and 7 months after treatment onset (T2) measured by CBCT and comparing CLB and SLB groups.

Measurements	SLB (G1) (n = 13)	CLB (G2) (n = 12 )	Diff.	P value
P1L BBT (mm)	-0.66 ± 1.65	-1.51 ± 1.84	0.85	0.234
P1R BBT (mm)	-0.88 ± 1.17	-0.90 ± 1.34	0.02	0.964
P1 TWBB (mm)	-0.56 ± 1.04	-0.21 ± 1.38	-0.35	0.475
P2L BBT (mm)	-0.64 ± 0.57	-0.88 ± 2.06	0.25	0.681
P2R BBT (mm)	-1.09 ± 0.83	-1.09 ± 1.16	0.00	0.995
P2 TWBB (mm)	-0.01 ± 1.10	-0.66 ± 1.76	0.65	0.275
M1L BBT (mm)	-0.32 ± 0.75	-0.31 ± 0.97	0.01	0.992
M1R BBT (mm)	-0.54 ± 0.77	-0.79 ± 0.85	0.25	0.452
M1 TWBB (mm)	-0.10 ± 1.15	-0.26 ± 1.72	0.16	0.537

M1 = first molar, P2 = second premolar and P1 = first premolar.

Mandibular buccal bone thickness (BBT) decreased from T_1_ to T_2_ for both bracket types. BBT in the CLB group significantly decreased for P1L (-1.51 mm; p = 0.016), P1R (-0.9 mm; p = 0.039), P2R (-1.09 mm; p = 0.007) and M1R (-0.79 mm; p = 0.008). BBT in the SLB group significantly decreased for P1R (-0.88 mm, p = 0.019), P2L (-0.64 mm; p = 0.002), P2R (-1.09 mm, p < 0.001) and M1R (-0.54 mm; p = 0.025). However, changes in TWBB measurements showed a slight decrease and were not considered statistically significant in either one of the groups: for the CLB group, the following measurements decreased: P1 (-0.21 mm; p = 0.613), P2 (-0.66 mm; p = 0.222) and M1 (-0.31 mm; p = 0.611); as for the SLB group, the following measurements decreased: P1 (-0.56 mm; p = 0.076) and P2 (-0.01 mm; p = 0.980), with an increase in M1 (0.10 mm; p = 0.750).

Comparison between BBT and TWBB measurements from T_1_ to T_2_revealed no significant differences between groups (Table 4). Additionally, no significant differences were found when comparing dental casts at treatment onset (T_1_) and 7 months later (T_2_) (Table 5). An average increase of dental transversal distances occurred from T_1_ to T_2_, which was considered significant. Bracket type had no significant influence on changes in mandibular dental arch. Differences between SLB and CLB for interfirst premolar width, intersecond premolar width and intermolar width were -0.6 mm (p = 0.489), 0.35 mm (p = 0.465) and 0.46 mm (p = 0.180), respectively.

**Table 5.** Means and standard deviation at the beginning of treatment (T1) and 7 months after treatment onset (T2) measured in dental casts and comparing CLB and SLB groups.

Measurements	SLB (G1) (n = 13)	CLB (G2) (n = 12 )	Diff.	p value
4-4 width	1.27 ± 1.95	1.87 ± 2.30	-0.60	0.489
5-5 width	2.10 ± 1.00	1.75 ± 1.33	0.35	0.465
6-6 width	0.92 ± 0.88	0.46 ± 0.77	0.46	0.180

Furthermore, no statistically significant association was found between transversal width of buccal bone (TWBB) and dental expansion (Table 6).

**Table 6.** Pearson correlation coefficient between transversal width of buccal bone (TWBB) and dental expansion within the two bracket system groups.

Measurements	r	P
P1 TWBB	0.15	0.467
P2 TWBB	0.28	0.176
M1 TWBB	0.09	0.676

M1 = first molar, P2 = second premolar and P1 = first premolar.

## DISCUSSION

In this sample, patients were treated by different dentists, but in order to obtain more reliable results, measurements were made by only one previously calibrated examiner. The error of the method used to assess intra and interexaminer reliability proved to be small. No significant differences were found between measurements made by two operators at two different time points. Interexaminer analysis showed that errors ranged from 0.30 to 1.08 mm. This may have occurred due to high resolution images offering excellent view without overlapping structures.

A disadvantage of the CBCT method is its greater radiation dose in comparison to conventional radiographs (periapical and panoramic). However, CBCT is an invaluable tool in orthodontic research. Good to excellent reliability of CBCT scans used for detection of bone defects was demonstrated by Misch *et al*.^23^ Furthermore, when compared to bidimensional radiographs, CBCT showed great reliability and offered advantages when detecting and quantifying bone fissures and fenestrations, as well as periodontal defects in the buccal bone.^24^

Mandibular arch bone expansion studies with CBCT scans comparing SLB and CLB are rare in the literature. And few studies have assessed the maxillary arch response to SLB and CLB systems.^19^ Nonetheless, some studies compared arch expansion on dental casts and on digitized models, which may offer great accuracy.^7,10,11^ Claims have been made that SLB can result in broader arch forms in comparison to CLB.^4^ Thus, this study aimed at testing the null hypothesis that there are no significant differences in the amount of expansion of the mandibular arch (dental and alveolar bone changes) during the first 7 months of alignment and leveling when either SLB or CLB systems are used, as demonstrated by analysis on CBCT and dental casts. 

According to Birnie,^25^ Damon divulged his theory that by using SLB with low friction and light forces more stable biological results could be produced. Damon,^4^ based on empirical and anedotical evidence, attributed advantages to self-ligating brackets, among which is the passive expansion of the arches. The Damon SLB system claims that post-treatment computed tomography images show transverse arch development and normal alveolar bone on buccal surface. Low friction and low force are purported to be good to physiologically rebuild the alveolar bone.^26^

The three-dimensional capability of CBCT makes it possible to noninvasively assess alveolar bone changes for mandibular posterior teeth. We found that BBT and TWBB measurements decreased from T_1_ to T_2_ for both groups. A significant difference occurred for the majority of measurements regarding BBT from T_1_ to T_2_ for both groups. There was significant difference for the following measurements, from T_1_ to T_2_, regarding BBT changes: CLB group - P1L (-1.51 mm, p = 0.016), P1R (-0.90 mm, p = 0.039), P2R (-1.09 mm, p = 0.007), M1R (-0.79 mm, p = 0.008); SLB group - P1R (-0.88 mm, p = 0.019), P2L (-0.64 mm, p = 0.002), P2R (-1.09 mm, p < 0.001), M1R (-0.54 mm, p = 0.025). However, no significant differences were found between groups. Furthermore, no significant differences from T_1_ to T_2_ were observed between and within groups for TWBB.

The results of the present study confirm findings in the literature showing similar behaviors for both brackets, particularly with regard to dental expansion assessed by means of dental casts. Mandibular arch alignment resulted in transverse expansion irrespective of the appliance system used. Interfirst premolar distances, measured on dental casts with a digital caliper in both groups, increased (SLB, 1.27 mm; CLB, 1.87 mm). This result is similar to those found by Fleming *et al,* 7 with an increase of 0.85 mm and 1.17 mm for SLB and CLB, respectively. However, the change was not significantly different between the two bracket systems. Further corroborating these findings, Vajaria el al^11^ also found expansion in interfirst premolar distances. As for intersecond premolar distances, there was an increase of 2.10 mm for SLB and 1.75 mm for CLB; however, this increase was similar for both groups. Once again, the results yielded by the present study are similar to those obtained by Fleming *et al* 7 (SLB= 1.43 mm, and CLB= 1.72 mm). Nevertheless, contrary to our findings, Vajaria *et al* 11 found a larger increase for the self-ligating group (4.35 mm in comparison to 2.6 mm for the conventional group). Regarding intermolar distances, there was an increase ranging from 1.4 to 2.4 mm for SLB, and from 0.43 to 1.85 mm for CLB.^7,9,10,11,17,27,28^ On the other hand, a decrease in intermolar distance was observed in only one study in which cases were treated by means of premolar extractions.^28^ We found nonsignificant increases of mandibular first intermolar width for both SLB and CLB groups, and there was no significant difference between the two bracket groups. The present study showed molar expansion of 0.92 mm and 0.46 mm for SLB and CLB, respectively. This result is in accordance with the study by Vajaria* et al.* 11 Nonetheless, Pandis *et al^*10*,*17*^* and Fleming *et al* 7 found that SLB expanded more than CLB in the molars region, and this difference was considered statistically significant.

When the Pearson correlation coefficient was assessed, we found that the alveolar buccal bone did not follow dental expansion. Therefore, the statements wherein self-ligating brackets produce physiological and passive movements of the arches were not confirmed in this study, at least 7 months after orthodontic treatment onset. Regarding buccal bone changes, it seems that self-ligating appliances do not offer any advantages over the conventional bracket system. Thus, the null hypothesis of the present study was accepted; in other words, no significant differences were found between self-ligating and conventional brackets systems regarding mandibular buccal bone plate expansion or dentoalveolar expansion.

## CONCLUSIONS

» There is no difference between patients treated with self-ligating brackets or conventional brackets, regarding mandibular dentoalveolar expansion.

» There is no difference between patients treated with self-ligating brackets or conventional brackets, regarding buccal bone plate changes (mandibular buccal bone thickness and transversal width of buccal bone).

» There were no significant correlations between buccal bone plate changes and dentoalveolar expansion within groups.
